# Ecologic association between influenza and COVID-19 mortality rates in European countries

**DOI:** 10.1017/S0950268820002125

**Published:** 2020-09-11

**Authors:** S. Petti, B. J. Cowling

**Affiliations:** 1 Department of Public Health and Infectious Diseases, Sapienza University, Rome, Italy; 2 WHO Collaborating Centre for Infectious Disease Epidemiology and Control, School of Public Health, Li Ka Shing Faculty of Medicine, The University of Hong Kong, Hong Kong, China

**Keywords:** COVID-19, influenza

## Abstract

Ecologic studies investigating COVID-19 mortality determinants, used to make predictions and design public health control measures, generally focused on population-based variable counterparts of individual-based risk factors. Influenza is not causally associated with COVID-19, but shares population-based determinants, such as similar incidence/mortality trends, transmission patterns, efficacy of non-pharmaceutical interventions, comorbidities and underdiagnosis. We investigated the ecologic association between influenza mortality rates and COVID-19 mortality rates in the European context. We considered the 3-year average influenza (2014–2016) and COVID-19 (31 May 2020) crude mortality rates in 34 countries using EUROSTAT and ECDC databases and performed correlation and regression analyses. The two variables – log transformed, showed significant Spearman's correlation *ρ* = 0.439 (*P* = 0.01), and regression coefficients, *b* = 0.743 (95% confidence interval, 0.272–1.214; *R*^2^ = 0.244; *P* = 0.003), *b* = 0.472 (95% confidence interval, 0.067–0.878; *R*^2^ = 0.549; *P* = 0.02), unadjusted and adjusted for confounders (population size and cardiovascular disease mortality), respectively. Common significant determinants of both COVID-19 and influenza mortality rates were life expectancy, influenza vaccination in the elderly (direct associations), number of hospital beds per population unit and crude cardiovascular disease mortality rate (inverse associations). This analysis suggests that influenza mortality rates were independently associated with COVID-19 mortality rates in Europe, with implications for public health preparedness, and implies preliminary undetected SARS-CoV-2 spread in Europe.

## Introduction

The COVID-19 outbreak, caused by the severe acute respiratory syndrome coronavirus 2, SARS-CoV-2, evolved in two distinct phases. The former was a local outbreak first detected in China in December 2019, and the latter the subsequent spread of the virus to the rest of the world. On 11 March 2020, the World Health Organization (WHO) made the assessment that the COVID-19 outbreak could be characterised as a pandemic [1]. In the beginning of the second phase, the COVID-19 pandemic has been particularly severe in Europe and North America. Indeed, by the end of May mortality rates were as high as 22.5 and 14.3 per 100 000, respectively, while in the remaining continents rates were lower than 1.0 per 100 000 [2]. COVID-19 spread in Europe has been uneven, with Italy experiencing the highest death toll in February and March, followed by other Western countries. By the end of May, COVID-19 mortality rates were ranging between higher than 50 per 100 000 in Belgium, Spain, UK and Italy, and lower than 2 per 100 000 in Slovakia, Greece and Bulgaria. Several factors probably explained these varying mortality rates, such as nature and timeliness of implementation of COVID-19 control policy measures [3], demographic variables [4], healthcare system quality and ability to manage the rapid COVID-19 spread [5–7] and the timing of SARS-CoV-2 introduction in the community [8].

As for the last issue, several studies suggest that SARS-CoV-2 could be circulating in Europe before the detection of the early COVID-19 cases. Namely, although the first Italian patient with COVID-19 was identified on 20 February 2020 and the earlier containment measures were already implemented on 21 February [9], on 21–29 February almost 3% of the residents of a small Italian town in the area of the outbreak epicentre resulted infected with SARS-CoV-2 [10]. In addition, seroprevalence of SARS-CoV-2 (test accuracy, 100% sensitivity, 98.2% specificity) in blood samples collected in December 2019 from healthy donors living in Milan was as high as 2.7%, according to a preprint survey [11], while a nasopharyngeal swab collected in December 2019 from a French patient admitted to intensive care unit for severe influenza-like illness (ILI) was reanalysed in April 2020 and resulted positive for SARS-CoV-2 [12]. Phylogenetic analyses also support this hypothesis, showing that SARS-CoV-2 was circulating outside China since fall 2019, and there have been multiple SARS-CoV-2 introductions in Europe [13–15]. Uncontrolled virus circulation in humans before its discovery is typical of the human coronavirus (HCoV) species [16]. The investigation of the dynamics of the COVID-19 outbreak must, therefore, account for SARS-CoV-2 circulation that occurred before the implementation of nationwide public health measures and that could help explain why SARS-CoV-2 was ubiquitous in Europe as early as in May, with seroprevalence estimates of 1–2% in blood donors and 4–5% in the general population, with peaks of 10–15% [17–19], that were likely limited by the unprecedented public health measures implemented in most countries after the detection of COVID-19 cases [3].

The design of specific anti-COVID-19 control measures, the implementation of community-based control strategies and the proper allocation of resources, can benefit from the investigation of the country-based determinants associated with COVID-19 mortality and severity. For this reason, during the SARS-CoV-2 pandemic many ecologic studies have been performed. The majority of them considered variables that reflected at population level the risk factors for COVID-19 severity at an individual level, such as population ageing, prevalence of diseases associated with COVID-19 death and severity, healthcare system capacity to face the public health emergency, etc. [4, 20–22]. The assessment of COVID-19 mortality determinants could benefit from similarities between this and other respiratory infectious diseases, particularly influenza, as these diseases share several characteristics. Indeed, during the 2019–2020 season, ILI and COVID-19-like illness (CLI) followed similar weekly incidence rate trends, although absolute values were different, as shown by the National Syndromic Surveillance Program in USA [23], and the general practitioners' network in France [24]. In addition, the public health measures taken to constrain the SARS-CoV-2 outbreak in Japan also limited the activity of seasonal influenza [25]. Similarity between influenza and COVID-19 incidence and mortality rates, however, does not result in the equivalence between these diseases, since influenza virus and SARS-CoV-2 are two distinct species of enveloped RNA virus belonging to two different families. Indeed, there are clinical, epidemiological and biological differences between the two diseases [26] which lead to differences in disease burden, case-fatality rates, proportion of asymptomatic individuals, etc. However, Tolksdorf and colleagues found that community-based influenza determinants could somewhat predict COVID-19 burden [27]. Thus, in addition to the aforementioned aggregated variables, population-level counterparts of individual-level COVID-19 severity risk factors, influenza-related variables could be eligible as determinant of COVID-19 mortality in European countries, to build an accurate COVID-19 mortality model.

Therefore, the aim of this study was to investigate the ecologic association between influenza and COVID-19 mortality rates in the European countries.

## Methods

### Data sources and parameters

Data on COVID-19 deaths in 34 European countries were gathered from the COVID-19 database of the European Centre for Disease Prevention and Control (ECDC) [2]. Crude COVID-19 mortality rates (number of deaths per 100 000) were assessed using the population on 1 January 2020 extracted from the EUROSTAT database [28]. This study focused on the first major epidemic waves of COVID-19 in Europe up until 31 May 2020 [29].

Crude influenza mortality rates for the last available years (i.e. 2014–2016), were assessed. The number of influenza deaths in a given year and the population on 1 January of that year were extracted from the EUROSTAT database [28] and crude mortality rates calculated. Then, for each country the 3-year average influenza mortality rates were assessed.

Some, but not all demographic, health and healthcare determinants, potentially associated with influenza and COVID-19 mortality rates also were extracted through the same database and assessed. Namely, 3-year average population (2014–2016), life expectancy at birth (2018), healthy life years at birth (2018, available only for a subset of countries), 3-year average crude all-cause mortality rate (per 1000, 2014–2016), 3-year average crude pneumonia mortality rate (per 100 000, 2014–2016), crude cardio-vascular disease (CVD) mortality rate (per 100 000, 2016), number of hospital beds per 100 000 (2017), 3-year average influenza vaccination coverage in population aged ≥65 years (2014–2016, available only for a subset of countries).

### Data analysis

The association between COVID-19 and 3-year average influenza mortality rates was explored using the nonparametric Spearman's correlation coefficient *ρ*. Then, the COVID-19 and 3-year average influenza mortality rates were log transformed to normalise variances and simple and multiple regression analyses were performed with log COVID-19 mortality rate as dependent variable. Zero values that could not be log transformed were given the lowest detected value.

The explanatory variables initially considered for the multiple regression analysis were, 3-year average population, life expectancy at birth, healthy life years at birth, 3-year average influenza vaccination coverage in population aged ≥65 years, 3-year average crude all-cause mortality rate, 3-year average crude pneumonia mortality rate, crude CVD mortality rate, number of hospital beds per population unit. Mortality rates, average population and number of hospital beds were log transformed. Correlation matrix was preliminarily performed to investigate collinearity that could inflate the coefficient estimates. Only non-correlated variables, with Pearson's correlation coefficients <0.4, were considered. Influenza mortality was forced into the model. In order to control the regression model for overfitting, due to the inclusion of unneeded predictors, the regression was initially run with all the non-collinear variables and variables that yielded statistically non-significant coefficient estimates (*P* ≥ 0.05) were excluded, thus obtaining a limited set of meaningful variables.

In order to study whether influenza and COVID-19 mortality rates shared common determinants that may help justify the similarity between these population-based variables, a series of simple regression analyses was designed treating both COVID-19 and influenza mortality rates as dependent variables, and using the same set of determinants considered for the multiple regression analysis.

Since the influenza mortality rates could be unreliable in small countries, the analysis was repeated considering only countries with population higher than 2 000 000 individuals.

The agreement between influenza and COVID-19 mortality severity also was studied. More specifically, countries were grouped in quartiles according to the two mortality rates, and the agreement between influenza and COVID-19 quartiles was investigated. The absolute agreement (i.e. the proportion of countries located in the same influenza and COVID-19 quartile), and the intraclass correlation coefficient (ICC), were assessed. Two-way absolute agreement single measure ICC was used, considering the country classification into COVID-19 mortality quartiles as reference value [30].

In order to explore the potential of a multivariate ecologic study to predict COVID-19 mortality (actually, this was not an aim of the current study), the agreement between the observed COVID-19 mortality and the COVID-19 mortality estimated by the multiple regression analysis, also was investigated using the same methodology.

## Results

There were 34 countries included in the analysis (Supplementary Table S1). The overall COVID-19 mortality rate was 27.76 per 100 000, the lowest and highest rates were reported in Slovakia and Belgium with 0.51 and 82.52 per 100 000, respectively. The overall 3-year average influenza mortality rate in the 34 countries was roughly 30 times lower, namely, 0.91 per 100 000, and the lowest and highest rates were reported in Lichtenstein and Finland, with 0.00 and 2.49 per 100 000, respectively. The two mortality rates were correlated (Spearman's *ρ* = 0.439; *P* = 0.01).

The simple regression coefficient was *b* = 0.743 (95% confidence interval, 0.272–1.214; *P* = 0.003), with *R*^2^ = 0.244 (Table 1), suggesting that 3-year average influenza mortality rate could explain 24.4% of the between-country variations in COVID-19 mortality rate.

**Table 1.** Simple and multiple regression analyses with crude COVID-19 mortality rate as dependent variable

	Coefficient	95% confidence interval	*P*
Simple regression			
Influenza mortality	0.743	0.272–1.214	0.003
Multiple regression – initial model			
Influenza mortality	0.561	0.140–0.982	0.01
Population	0.336	0.144–0.529	0.001
Pneumonia mortality	0.413	−0.200–1.027	0.17
CVD mortality	−0.935	−1.687 to −0.182	0.01
Multiple regression – final model			
Influenza mortality	0.472	0.067–0.878	0.02
Population	0.351	0.157–0.545	0.007
CVD mortality	−1.043	−1.788 to −0.299	<0.0001

All variables were log transformed.

Influenza mortality, 3-year average crude influenza mortality rate; population, 3-year average population; pneumonia mortality, crude pneumonia mortality rate; CVD mortality, crude cardio-vascular disease mortality rate.

Simple regression *R*^2^ = 0.244; multiple regression initial model *R*^2^ = 0.576; final model *R*^2^ = 0.549.

Several investigated determinants were highly inter-correlated (Supplementary Table S2), and after the elimination of collinear variables, four variables were remaining that were considered for the initial multiple regression model (Table 1). After the removal of pneumonia mortality rate, the regression coefficient for influenza mortality resulting from the final model was *b* = 0.472 (95% confidence interval, 0.067–0.878; *P* = 0.02), with final model *R*^2^ = 0.549 (Table 1), that confirmed the robustness of the association between the two mortality rates.

Life expectancy at birth, influenza vaccination coverage in the elderly (direct associations), number of hospital beds and CVD mortality rates (inverse associations) were significantly associated with both influenza and COVID-19 mortality rates, while population size was directly associated with COVID-19 mortality (Table 2).

**Table 2.** Associations between demographic, health and healthcare determinants and 3-year average crude influenza mortality rate and crude COVID-19 mortality rate (log transformed), assessed through simple regression analyses (regression coefficients; 95% confidence intervals in brackets)

Determinant	Influenza mortality rate	COVID-19 mortality rate
3-year average population 2014–2016^a^	0.05 (−0.13 to 0.24)	0.37 (0.12–0.61)^b^
Life expectancy at birth 2018	0.07 (0.03–0.12)^b^	0.12 (0.06–0.18)^b^
Healthy life expectancy at birth 2018^c^	0.01 (−0.02 to 0.03)	0.03 (−0.01 to 0.07)
3-year average crude mortality rate 2014–2016^a^	−0.17 (−1.31 to 0.96)	−0.83 (−2.50 to 0.85)
3-year average crude pneumonia mortality rate 2014–2016^a^	−0.38 (−0.92 to 0.16)	0.35 (−0.49 to 1.18)
Crude cardiovascular disease mortality rate 2016^a^	−0.64 (−1.26 to −0.02)^b^	−1.29 (−2.18 to −0.41)^b^
Hospital beds × 100 000 population 2017^a^	−0.70 (−1.49 to −0.10)^b^	−1.22 (−2.40 to −0.04)^b^
3-year average influenza vaccination population ≥65 years (2014–2016)^d^	0.01 (0.002–0.01)^b^	0.02 (0.01–0.02)^b^

a Log transformed.

b *P* < 0.05.

c Available for 32 countries.

d Available for 31 countries.

The countries with population lower than 2 000 000 were Lichtenstein, Iceland, Malta, Luxembourg, Cyprus, Latvia and Estonia. The analyses repeated considering only the remaining 27 countries improved the association between influenza and COVID-19 mortality rates, and confirmed the previous results. Namely, Spearman correlation *ρ* = 0.476 (*P* = 0.01), simple regression coefficient *b* = 0.837 (95% confidence interval, 0.326–1.349; *P* = 0.002; *R*^2^ = 0.313), multiple regression coefficient *b* = 0.887 (95% confidence interval, 0.438–1.336; *P* = 0.0004; *R*^2^ = 0.496) (data not shown in table).

Ten countries were classified in the same COVID-19 and influenza mortality quartiles, with a fair absolute agreement of 29.4%, that was higher for countries in the first and the highest quartiles. Namely, Bulgaria, Cyprus, Lichtenstein and Slovakia were in the first quartiles, and Belgium, France, Netherlands, Sweden were in the fourth quartiles (Supplementary Table S3). Seventeen countries showed a discrepancy of only one quartile, while two-quartile discrepancies were reported for seven countries and no third-quartile discrepancy was found. The ICC resulted 0.44 (95% confidence interval, 0.12–0.68).

The highest COVID-19 mortality rates estimated through multiple regression were provided for France, Germany, Spain, Italy and UK, while the lowest were provided for Lichtenstein, Slovakia, Hungary, Bulgaria and Cyprus (Supplementary Table S4). As expected, the multiple regression model provided higher agreement between quartile distributions. Indeed, the absolute agreement was 55.9% (19 countries) and ICC = 0.723 (95% confidence interval, 0.512–0.852) (Supplementary Table S3).

## Discussion

This analysis showed that 3-year average influenza mortality rate was associated with COVID-19 mortality rate in the European context, although influenza mortality alone could explain only part of the COVID-19 mortality variability. The discrepancy between the two mortality rates was likely due to the aforementioned differences between the two diseases at population and individual levels [26, 31].

An apparently perplexing characteristic of the reported association between the two mortality rates was that while influenza virus circulation during the seasons considered in the present analysis was uncontrolled, SARS-CoV-2 circulation was probably limited by the widespread exceptional public health measures implemented in Europe [32]. Therefore, assuming that the reported association between the two rates was not spurious, the most likely explanation of the present results was that SARS-CoV-2 circulation also was partly uncontrolled. Actually, surveys and phylogenetic analyses support the idea of multiple introductions of the virus in Europe since 2019 [10–15]. Such an undetected virus circulation is not surprising, since patients with COVID-19/CLI have been frequently misclassified as patients with ILI [23, 24], and is corroborated by SARS-CoV-2 seroprevalence surveys [17–19, 33]. The implementation of country-based control policies likely prevented further severe SARS-CoV-2 outbreak propagation, thus explaining the COVID-19 incidence rate of 3–5% in May 2020, lower than influenza rate that is usually 10% or higher [34, 35]. The history of other HCoVs corroborates this hypothesis. For example, the first patient infected with HCoV-NL63, a child with atypical bronchiolitis, was detected in January 2003 in Amsterdam. Soon after, HCoV-NL63 positive patients from all over the world with upper and/or lower respiratory tract infections were detected, and seroprevalence values as high as 2–9% were reported. Such an apparently rapidly spreading pandemic was explained by the long undetected virus circulation confirmed by the analysis of a specimen collected from a child with pneumonia that was stored on kidney simian cells since 1988. Thus, HCoV-NL63 was already circulating fifteen years before its detection [16, 36].

Relatively free SARS-CoV-2 circulation in Europe also was promoted by inefficient and untimely crisis coordination at central level [29], and by delays and contradictions of some international public health organisations in acknowledging community transmission, typical of pandemics, that must lead to public health control measures. Indeed, on 19 April 2020, community transmission was not acknowledged yet in France, Spain, UK, Italy, where 15 000–25 000 COVID-19 deaths were already reported, and in Belgium and the Netherlands, with 3500–5000 deaths, but was confirmed in small countries such as San Marino, Andorra, Bosnia and Kosovo [37]. The question remains unanswered, on whether earlier community transmission acknowledgement in Europe, and consequent timely implementation of coordinated COVID-19 control measures would have limited the high burden of COVID-19.

The current study corroborated the assumption that influenza and COVID-19 mortality rates share similar determinants. Indeed, both diseases were significantly associated with similar demographic, health and healthcare determinants, excluding population size that was associated only with COVID-19 (Table 2). This is also the reason why crude mortality rates were used instead of standardised rates, as the standardisation process would have reduced the impact of population age structure on mortality rates, while the rationale of the current study was that influenza and COVID-19 share similar population-based determinants, and population age structure was among them.

Influenza and COVID-19 share another important population-level characteristic. Namely, the problem of misclassifications and disagreements in classification that lead to inconsistent burden of disease estimates. Although influenza has been recognised as an important cause of mortality, particularly in the elderly, mortality rates are generally low, because much of related mortality is not attributed to primary influenza infection, but to complications and secondary infections. This problem generated incongruences in classifying influenza as underlying or contributing cause of death [38]. As for COVID-19, differences in mortality between countries and even within countries were partly attributable to the use of different criteria to classify COVID-19 deaths [39]. To overcome the problem of misclassification the US National Center for Health Statistics coined an aggregated variable called ‘PIC’, that considered all deaths attributed to pneumonia, influenza and COVID-19, updating another variable called ‘P&I’, based on influenza and pneumonia [40]. In the current study, however, pneumonia mortality did not result associated with influenza and COVID-19 (Table 2), and unreported analyses using 3-year average ‘P&I’ mortality rate provided non-significant results.

Influenza and COVID-19 mortality rates resulted associated with population age structure, as shown in Table 2, and corroborated by the EUROSTAT report showing that between 2012 and 2016, as many as 70% influenza deaths occurred in the elderly aged ≥65 years, and the European standardised influenza mortality rates in this age group were between ten and twenty times higher than in subjects younger than 65 years [41]. The COVID-19 burden in the elderly was even higher. Indeed, the elderly aged ≥65 years accounted for 90–95% of deaths in European countries and their risk of dying was up to 80 times higher than in younger individuals [42]. Another characteristic shared by influenza and COVID-19 deaths was the impact of comorbidities on mortality. Indeed, three-fourth influenza deaths occur in patients with comorbidities [43], while for COVID-19 such a proportion is higher than 90% [42, 44].

This study reported an inverse association between number of hospital beds and mortality rates (Table 2), thus showing that high influenza and COVID-19 mortality was also due to inefficiencies of the healthcare systems, and corroborated by data from several European countries [45]. Similarly, the inadequateness of the healthcare system has been responsible for the high COVID-19-related death toll reported in many countries, such as UK [5], Italy [46] and Spain [47].

The direct association between influenza vaccination coverage among the elderly and influenza and COVID-19 mortality rates reported in this study (Table 2) was corroborated by population-based studies, and the Cochrane systematic review focusing on the efficacy of influenza vaccination in the elderly showing an unclear effect on improving mortality [48–50]. This paradoxical effect of influenza vaccine is due to the fact that vaccine uptake is more likely in the categories who need it least, that is, women, elderly younger than 80 years and subjects without comorbidities [51], an effect called Inverse Care Law by Julian Tudor Hart in 1971, who stated that ‘The availability of good medical care tends to vary inversely with the need for it in the population served’ [52]. Unfortunately, the Inverse Care Law also applies to preventive medicine including cancer screening [53, 54], and influenza vaccination [55], and explains the apparently puzzling direct association between influenza vaccination coverage and COVID-19 mortality, since COVID-19 mortality risk was twofold higher in men than in females, 13-fold higher in individuals older than 80 years than in those aged 65–79 years, and 5-to-15-fold higher in patients with comorbidities, than in those without [56]. In other words, individuals at higher influenza and COVID-19 mortality risk are those who are less likely to get vaccinated. The reported association between high influenza vaccine coverage and high influenza and COVID-19 mortality has nothing to do with intrinsic vaccine efficacy, since ecologic studies are subject to ecologic fallacy that prevents from inferring associations at an individual level.

The multiple regression analysis showed that CVD mortality was inversely associated with COVID-19 mortality (Table 1). CVD, particularly ischaemic heart disease and stroke, is the leading cause of death in Europe, accounting for 40% and 49% of all deaths in males and females, respectively, and is also the leading cause of premature death, accounting for more than 35% of all deaths under 75 years. Differences in CVD mortality are, therefore, the main responsible of differences in life expectancy at birth, country distribution for age, and potential years of life lost. These differences are particularly evident between Eastern and Western European countries [57]. CVD and older age are also the main risk factors associated with COVID-19 death at an individual level [58–60].

These considerations help explain why Western European countries showed generally high influenza and COVID-19 mortality rates, while Eastern European countries showed the reverse. Indeed, COVID-19 and influenza are particularly lethal in elderly individuals and, thus, influenza and COVID-19 mortality rates are particularly high in countries where the proportion of elderly is higher. Since CVD deaths are responsible for premature mortality, countries where CVD mortality is higher, also are those with the lowest proportion of elderly and, consequently, with the lowest proportion of susceptible individuals at higher risk of dying from both COVID-19 and influenza.

Present research is an ecologic study with all the corresponding advantages and disadvantages of this approach. Indeed, the use of aggregated data prevented the identification of associations at an individual level, a problem known as ecologic fallacy. On the other hand, since these studies are relatively simple and reproducible they provide useful information in emergency situations like the COVID-19 pandemic. Such information, however, must be considered carefully and implementing public health control measures on the basis of ecologic studies alone could be problematic [61]. During this pandemic several ecologic studies have been published, reporting associations between COVID-19 incidence and mortality rates and BCG vaccine coverage [62], malaria prevalence [63], environmental and meteorological factors, pollutants (reviewed in [64]), etc. Although these associations were robust enough, they could not be considered to design COVID-19 control policies, due to ecologic fallacy. In the same way, the current study did not show that influenza prevention at an individual level leads to COVID-19 prevention, but only that the two mortality rates were associated at the population level.

The second important limit of this study was the reported problem of the reliability of both influenza and COVID-19 death counts [38, 39], that could lead to uncertainties in the true mortality rates in the countries under investigation in this study. An ideal, yet unfeasible, approach would be that every dying individual with ILI, CLI, acute respiratory illness and pneumonia was tested for all the circulating influenza strains and for SARS-CoV-2. In the case of influenza, this uncertainty regarding the death counts, led to varying estimates of the global number of attributable deaths, ranging from the lowest limit provided by the Global Burden of Disease Study of 99 000, to the highest limit provided by the CDC of 650 000 [65]. The problem of consistency of aggregated data, however, is shared by almost all diseases and conditions. For example, the estimated global number of deaths from breast cancer was 630 000 according to GLOBOCAN 2018 [66], and 535 000 according to the Global Burden of Disease Study [67], with important differences within each country.

The last limitation of this study was that influenza mortality rate alone could not be considered an optimal COVID-19 mortality rate predictor, since the multiple regression analysis showed that there were other important population-based confounders associated with COVID-19 mortality. They could be variables related to age structure and prevalence of comorbidities associated with COVID-19 mortality. For example, age structure explained part of the between-country differences in COVID-19 mortality and case-fatality rates [4, 20]; median prevalence of the five conditions most frequently associated with severe COVID-19 in USA allowed to identify the areas at highest risk for COVID-19 death [21]; age-specific prevalence of comorbidities explained the differences in mortality between Nigeria, Brazil and Italy [22]. Economic and healthcare associated variables are other aggregated data potentially useful to predict COVID-19 severity and spread [68–70], as well as inequalities within the general population [71]. Unlike these studies, however, the present analysis considered the mortality rate from an infectious disease that was not somewhat causally associated with COVID-19 mortality and death and was based on a different assumption, namely, that the two diseases shared a set of determinants, ranging from the characteristics of the population at highest risk, to transmission routes, from case and death misclassifications, to the efficiency of the healthcare systems.

In conclusion, influenza and COVID-19 mortality rates were significantly associated and influenza mortality could be an eligible predictor for the design of more accurate multivariable COVID-19 mortality assessment and prediction models.

## Data Availability

The data that support the findings of this study can be downloaded from the ECDC and EUROSTAT databases and are, in part, displayed in Supplementary material.

## Supplementary material

For supplementary material accompanying this paper visit http://dx.doi.org/10.1017/S0950268820002125. click here to view supplementary material
