# Supplementary material for: Ecologic association between influenza and COVID-19 mortality rates in European countries
Source: Epidemiol Infect. 2020 Sep 11;148:e209. doi: 10.1017/S0950268820002125 (PMC7506171; doi:10.1017/S0950268820002125)
Supplement: Supplementary file 1 [file S0950268820002125sup001.docx]

Epidemiology and Infection

Ecologic association between influenza and COVID-19 mortality rates in European countries

Stefano Petti, Benjamin J Cowling

Supplementary Material

**Supplemental Table 1**. COVID-19 (31 May 2020) and 3-year average influenza (years 2014-2016) crude mortality rates (per 100,000 population) in 34 European countries. Statistical analyses of association between mortality rates.

| Country | COVID-19 crude mortality rate | 3-year average crude influenza mortality rate |
| --- | --- | --- |
|  |  |  |
| Belgium | 82.52 | 1.43 |
| Bulgaria | 2.00 | 0.17 |
| Czechia | 3.00 | 0.91 |
| Denmark | 9.83 | 0.65 |
| Germany | 10.24 | 0.49 |
| Estonia | 5.06 | 1.37 |
| Ireland | 33.66 | 0.75 |
| Greece | 1.63 | 0.81 |
| Spain | 57.79 | 1.12 |
| France | 42.93 | 1.62 |
| Croatia | 2.53 | 0.67 |
| Italy | 55.24 | 0.69 |
| Cyprus | 1.94 | 0.27 |
| Latvia | 1.25 | 1.18 |
| Lithuania | 2.51 | 0.39 |
| Luxembourg | 17.92 | 0.77 |
| Hungary | 5.36 | 0.15 |
| Malta | 1.42 | 0.44 |
| Netherlands | 34.43 | 1.54 |
| Austria | 7.54 | 0.70 |
| Poland | 2.79 | 0.12 |
| Portugal | 13.58 | 0.83 |
| Romania | 6.45 | 0.16 |
| Slovenia | 5.19 | 0.79 |
| Slovakia | 0.51 | 0.12 |
| Finland | 5.73 | 2.49 |
| Sweden | 42.96 | 2.08 |
| United Kingdom | 57.58 | 0.55 |
| Iceland | 2.80 | 1.01 |
| Liechtenstein | 2.61 | 0.00 |
| Norway | 4.43 | 1.86 |
| Switzerland | 19.38 | 1.56 |
| Serbia | 3.48 | 0.37 |
| Turkey | 5.51 | 0.31 |
|  |  |  |
| Total | 27.76 | 0.94 |

Spearman’s correlation, ρ=0.439; p=0.01.

**Supplemental Table 2**. Correlation matrix between the set of explanatory variables considered for the multiple regression model

|  | Population | Life exp | Healthy life | All-cause | Pneumonia | CVD | Hosp beds | Vaccine | Influenza |
| --- | --- | --- | --- | --- | --- | --- | --- | --- | --- |
| Population | 1.000 |  |  |  |  |  |  |  |  |
| Life exp | -0.008 | 1.000 |  |  |  |  |  |  |  |
| Healthy life | 0.121 | 0.461 | 1.000 |  |  |  |  |  |  |
| All-cause | 0.313 | -0.634 | -0.422 | 1.000 |  |  |  |  |  |
| Pneumonia | 0.065 | -0.066 | 0.105 | -0.074 | 1.000 |  |  |  |  |
| CVD | 0.040 | -0.834 | -0.430 | 0.862 | -0.102 | 1.000 |  |  |  |
| Hosp beds | 0.167 | -0.585 | -0.337 | 0.639 | -0.072 | 0.668 | 1.000 |  |  |
| Vaccine | 0.162 | 0.792 | 0.514 | -0.389 | 0.025 | -0.704 | -0.595 | 1.000 |  |
| Influenza | 0.108 | 0.521 | 0.096 | -0.055 | -0.244 | -0.347 | -0.300 | 0.448 | 1.000 |

| Variable | Explanation |
| --- | --- |
|  |  |
| Population | 3-year average population (years 2014-2016, log transformed) |
| Life exp | Life expectancy at birth (last available year, 2018) |
| Healthy life | Healthy life years at birth (last available year, 2018) |
| All-cause | 3-year average crude all-cause mortality rate (per 1,000; years 2014-2016; log transformed) |
| Pneumonia | 3-year average crude pneumonia mortality rate (per 100,000; years 2014-2016; log transformed) |
| CVD | Crude cardiovascular disease mortality rate (per 100,000; year 2016; log transformed) |
| Hosp beds | Number of hospital beds (per 100,000; last available year, 2017; log transformed) |
| Vaccine | 3-year average influenza vaccination coverage in population aged ≥65 years, 2014-2016 (percent; available only for a subset of countries) |
| Influenza | 3-year average crude influenza mortality rate (per 100,000; years 2014-2016; log transformed) |

**Supplemental Table 3**. Distributions of countries in quartiles according to the observed crude COVID-19 mortality rate, 3-year average influenza mortality rate, and estimated crude COVID-19 mortality rates, as predicted by the multiple regression analysis displayed in Table 2. Countries are listed in alphabetical order, in bold are displayed correctly predicted countries. Statistical analysis of agreement between quartiles using the observed COVID-19 mortality quartiles as reference values, absolute agreement and Intraclass Correlation Coefficient (ICC; 95% confidence intervals in brackets)

| Observed COVID-19 mortality | 3-year average influenza mortality | Estimated by multiple regression |
| --- | --- | --- |
|  |  |  |
| First Quartile | | |
|  |  |  |
| Bulgaria  Croatia  Cyprus  Greece  Latvia  Liechtenstein  Lithuania  Malta  Slovakia | **Bulgaria**  **Cyprus**  Hungary  **Liechtenstein**  Poland  Romania  Serbia  **Slovakia**  Turkey | **Bulgaria**  Hungary  **Latvia**  **Liechtenstein**  **Lithuania**  **Malta**  Romania  Serbia  **Slovakia** |
|  |  |  |
| Second Quartile | | |
|  |  |  |
| Czechia  Estonia  Hungary  Iceland  Norway  Poland  Serbia  Slovenia | Austria  Croatia  Denmark  Germany  Italy  Lithuania  Malta  UK | Austria  Croatia  Cyprus  **Estonia**  **Iceland**  Luxembourg  **Poland**  **Slovenia** |
|  |  |  |
| Third Quartile | | |
|  |  |  |
| Austria  Denmark  Finland  Germany  Luxembourg  Portugal  Romania  Switzerland  Turkey | Czechia  Greece  Iceland  Ireland  Latvia  **Luxembourg**  **Portugal**  Slovenia  Spain | Czechia  **Denmark**  **Finland**  **Germany**  Greece  Ireland  Italy  **Portugal**  Sweden |
|  |  |  |
| Furth Quartile | | |
|  |  |  |
| Belgium  France  Ireland  Italy  Netherlands  Spain  Sweden  UK | **Belgium**  Estonia  Finland  **France**  **Netherlands**  Norway  **Sweden**  Switzerland | **Belgium**  **France**  **Netherlands**  Norway  **Spain**  Switzerland  Turkey  **UK** |
|  |  |  |

Statistical analysis

|  | Absolute agreement | | | | | ICC |
| --- | --- | --- | --- | --- | --- | --- |
|  | All quartiles | First quartile | Second quartile | Third quartile | Fourth quartile |  |
|  |  |  |  |  |  |  |
| 3-year average influenza mortality | 29.4%  (10/34) | 44.4%  (4/9) | 0.0%  (0/8) | 22.2%  (2/9) | 50.0%  (4/8) | 0.442  (0.121-0.678) |
| multiple regression | 55.9%  (19/34) | 66.7%  (6/9) | 50.0%  (4/8) | 50.0%  (4/8) | 62.5%  (5/8) | 0.723  (0.512-0.852) |

**Supplemental Table 4**. Estimated crude COVID-19 mortality rates, as predicted by the multiple regression analysis displayed in Table 2

| Country | Estimated mortality rate |
| --- | --- |
|  |  |
| Belgium | 17.53 |
| Bulgaria | 1.41 |
| Czechia | 7.98 |
| Denmark | 11.61 |
| Germany | 13.37 |
| Estonia | 3.41 |
| Ireland | 13.15 |
| Greece | 8.49 |
| Spain | 26.87 |
| France | 43.20 |
| Croatia | 4.06 |
| Italy | 16.28 |
| Cyprus | 4.12 |
| Latvia | 2.75 |
| Lithuania | 1.89 |
| Luxembourg | 5.61 |
| Hungary | 2.34 |
| Malta | 3.21 |
| Netherlands | 24.54 |
| Austria | 7.79 |
| Poland | 4.83 |
| Portugal | 11.04 |
| Romania | 2.61 |
| Slovenia | 4.98 |
| Slovakia | 2.62 |
| Finland | 12.98 |
| Sweden | 16.36 |
| United Kingdom | 23.59 |
| Iceland | 5.22 |
| Liechtenstein | 0.75 |
| Norway | 19.20 |
| Switzerland | 17.46 |
| Serbia | 2.72 |
| Turkey | 21.92 |
